# *Leishmania infantum *leishmaniasis in corticosteroid – treated patients

**DOI:** 10.1186/1471-2334-6-177

**Published:** 2006-12-18

**Authors:** Silvia Pittalis, Emanuele Nicastri, Francesco Spinazzola, Piero Ghirga, Michele De Marco, Maria Grazia Paglia, Pasquale Narciso

**Affiliations:** 1 National Institute for Infectious Diseases IRCCS L. Spallanzani Rome Italy

## Abstract

**Background:**

The number of leishmaniasis cases associated with immunosuppression has increased regularly over the past 20 years. Immunosuppression related to HIV infection, immunosuppressive treatment, organ transplantation, and neoplastic diseases increases the risk for *Leishmania*-infected people to develop visceral illness.

**Case presentation:**

Three cases of *Leishmania infantum *leishmaniasis in corticosteroid (CS)-treated patients are reported: an isolated lingual leishmaniasis in a farmer treated with CS for asthma, a severe visceral leishmaniasis associated with cutaneous lesions in a woman with *myasthenia gravis*, and a visceral involvement after cutaneous leishmaniasis in a man receiving CS.

**Conclusion:**

Physicians should recognise CS-treated patients as a population likely to be immunesuppressed. In immunodeficiency conditions, unusual forms of leishmaniasis can develop and foster the risk of a diagnostic delay and of poor response to therapy.

## Background

The number of leishmaniasis cases associated with immunosuppression has increased regularly over the past 20 years. Immunosuppression related to HIV infection, drugs, organ transplantation, and neoplastic diseases increases the risk for *Leishmania*-infected people to develop visceral illness. In southwestern Europe visceral leishmaniasis (VL) is the clinical form most frequently associated to immunosuppression, particularly to HIV infection [1,2]. In immunodeficiency conditions, unusual forms of leishmaniasis can develop and foster the risk of a fatal diagnostic delay and of a poor response to therapy.

## Case presentation

### Patient 1

In August 2001, a 79-year-old man was admitted to a community hospital because of a violaceous, thickened, dyskeratosic and painless lesion (diameter 2 × 2 cm) on his tongue with a deep central ulcer. A biopsy of the lesion showed deep inflammatory infiltration with macrophages containing numerous *Leishmania *amastigotes.

The patient, a farmer living in the rural area around Rome (Italy), was referred to our Institute. He had a glucose-6-phosphate dehydrogenase deficiency, suffered from asthma, and was given chronic corticosteroid (CS) therapy. The laboratory data on admission are reported in Table 1. Protein electrophoresis showed a mild hypergammaglobulinaemia. The immunofluorescence assay (Diamedix Corporation^®^, Miami-Florida) for the detection of anti-*Leishmania infantum *antibodies was positive (1:640). The search for antibodies against HIV resulted negative. A bone marrow aspirate did not show *Leishmania *amastigotes and a restriction fragment lenght polymorphism polymerase chain reaction (PCR-RFLP) [3] for the search of *Leishmania *DNA on bone marrow and peripheral blood resulted negative. *L. infantum *was identified by PCR-RFLP on the tongue biopsy.

**Table 1:** Laboratory values on admission

	**Patient 1**	**Patient 2**	**Patient 3**	**Normal value**
**Hemoglobin (g/dL)**	12.70	9.60	10	12–18
**White-cell count (per mm^3^)**	6000	16800	6000	4.3–10.8
**Neutrophils (%)**	63.30	93.80	66.60	40–75
**Lymphocytes (%)**	26.50	3.20	18.50	20–51
**Erythrocyte sedimentation rate (mm/h)**	35	90	65	<20
**CD4 cell count (per mm^3^)**	502	215	661	500–1200
**Platelet count (per mm^3^)**	227000	386000	514000	200000–400000
**Creatinine (mg/dL)**	0.7	0.12	1.78	0.5–1.4
**Protein (g/dl)**	7	4.9	6.6	6.2–8.2
**Albumin (%)**	57.7	50.3	56.4	52–65
**Gamma globulin (%)**	20.8	12	16.4	10–19
**Aspartate aminotransferase (U/L)**	20	14	21	<40
**Alanine aminotransferase (U/L)**	14	25	18	<40
**Alkaline phosphatase (U/L)**	59	91	322	<65
**Lactate dehydrogenase (mU/mL)**	360	244	458	266–500

The patient was treated with liposomal amphotericin B (3 mg/kg daily i.v. on days 1–5, 14 and 21) with complete recovery. After a 3-year follow-up, no relapse was noted.

### Patient 2

In February 2004, a 72-year-old housewife complained of weight loss, recurrent fever, and diarrhea after pneumonia. She lived in the rural area around Rome (Italy) and had been taking CS and myorelaxants for 16 years because of *myasthenia gravis*. In the year 2000, the patient was treated for visceral leishmaniasis with bone marrow involvement. A course of liposomal amphotericin B (1 mg/kg daily for 10 days) was repeated one year later for a relapse with involvement of bone marrow and duodenum: a clinical remission of fever and diarrhea was obtained.

On admission to our Institute, the patient was taking prednisone 25 mg daily and pyridostigmine 60 mg four times daily. She was febrile (38.5°C) with an impaired nutritional status (35 Kg) and a severe muscular hypotrophy. Upon examination, a first trigeminal branch herpes zoster, and mild hepatomegaly were found; the left nostril presented a cutaneous squamous and crusty lesion that had appeared 4 months earlier.

The results of hematochemical assays are shown in table 1; particularly, a severe lymphopenia (lymphocytes 538/mm^3^) with 215 CD4+ lymphocytes/mm^3 ^and 132 CD8+/mm^3 ^was found. HIV antibody test was negative. The chest X-rays showed an evolution from a single right basal consolidation to multiple bilateral pneumonic foci. The anti-*Leishmania infantum *serologic titer was 1:640 and the bone marrow biopsy showed numerous amastigotes of *Leishmania spp*. Intra-macrophagic amastigotes were also seen at histologic examination of the nasal lesion. *L. infantum *was identified by PCR-RFLP on peripheral blood and on nasal lesions. The patient was treated with liposomal amphotericin B (3 mg/kg daily for 10 days, total 30 mg/kg), and with levofloxacin (500 mg daily for 10 days), obtaining clinical remission with a gradual improvement of the nasal lesion. No relapse was reported during a 9-month follow-up.

#### Patient 3

A 54-year-old male, a farmer living in the rural area around Grosseto, Tuscany (Italy), was admitted to our Institute in March 2004 with a relapse of visceral and mucocutaneous leishmaniasis. In 1997 he complained of edema of the lips with maculopapular, non-itching lesions: a Miescher's granulomatous cheilitis was diagnosed and he was treated with local and systemic CS courses. The lesions of the perioral region disappeared, unlike the lips edema. In April 2003, he was admitted to a community hospital with remittent fever (40°C) associated to increasing asthenia and normocytic anemia. Amastigotes of *Leishmania *were recognized in the bone marrow and lip biopsies. The patient was treated with liposomal amphotericin B (3 mg/kg daily i.v. on days 1–5, 14 and 21, total 21 mg/kg). The fever disappeared and the edema of the lips substantially decreased. However, few weeks after the end of the therapy febrile episodes recurred, and in December 2003 a new course of amphotericin B therapy was prescribed (liposomal amphotericin B, 5 mg/kg daily i.v. for 10 days). Clinical improvement with persistent detection of *L. infantum *PCR-RFLP on peripheral blood was observed.

On admission to our Institute the patient was febrile (39°C) and presented lips edema with erythematous and cutaneous squamous lesions, diffuse lymphadenopathy and hepatosplenomegaly. The results of blood tests are shown in the table 1. The patient experienced a progressive reduction of hemoglobin (8.5 g/dL) and received two whole blood transfusions. Multiple blood cultures, HIV and Parvo virus B19 antibody searches were all negative. The anti-*Leishmania infantum *antibodies were positive (1:640), while the PCR-RFLP on peripheral blood resulted negative. The patient was treated with two subsequent 28-day courses of meglumine antimonate (20 mg/kg daily): he experienced a rapid clinical improvement and no adverse reactions. After a 6-month follow-up the patient is still in good condition, although a modest lip edema still remains. The antibody titer for *Leishmania infantum *is 1:160. The PCR for *Leishmania *spp. on peripheral blood is negative. The bone marrow biopsy shows no evidence of infection.

## Conclusion

Visceral leishmaniasis is a potentially fatal infection in immunocompromised hosts and current therapies frequently fail to eradicate *L. donovani *from infected tissue [4]. The clinical outcome is determined by the Th1 immune response, inducing the production of IFN-γ and IL-2 in response to leishmanial antigens [5,6].

Glucocorticoids affect the effector, suppressor, and cytotoxic T cells functions trough the blockade of cytokine expression [7], with the result of an increased susceptibility to infections, particularly with intracellular microbes [8] such as occurs with *Leishmania species*. In a murine model the prolonged use of steroids has been associated to a decreased production of IL-2, IFN-gamma, IL-4 and TNF-alfa and to a significant 3-fold increase in amastigote burden in the spleen [9].

We have presented 3 cases of CS-treated patients with diagnosis of *Leishmania infantum *leishmaniasis that posed serious clinical dilemmas in terms of diagnostic delay and partial response to therapy. Our three patients lived in rural areas of central Italy (Latium and Tuscany regions) where both visceral and cutaneous leishmaniasis are endemic. In the past 7 years, some 200 cases of visceral leishmaniasis were recorded from these regions, and in most of the patients parasites have been identified as *L. infantum *by means of isoenzyme analysis (unpublished data from Istituto Superiore di Sanità, Rome). Our first case is a paradigmatic description of the classical lip leishmaniasis that occurred over centuries in Southern Europe. The finding of a viscerotropic *Leishmania *as cause of a localized leishmaniasis poses the question whether the lesions observed may represent the site of the parasite inoculation or a secondary localization [10]. The absence of clinical, immunological and parasitological evidence of generalized parasite dissemination in the first case strongly supports the former hypothesis rather than secondary spread from an initial unknown source. Nevertheless the use of steroids and the elderly age of the patient could suggest a secondary involvement of the tongue [10]. The prolonged incubation period already described in few muco-cutaneous leishmaniasis cases [11,12] could suggest as primary option the endogenous reactivation of the infectious process promoted by the CS-induced impairment of the immune response. Particularly, a laryngeal leishmaniasis case has been described in a UK patient undergoing inhalation and oral steroid therapy for asthma who frequently visited Southern Europe without extra European travels [11].

In the further two cases the prolonged steroid use was likely to be associated to the clinical severity of the disease [13,14]. In the female patient affected by myasthenia, the relapses, the clinical spread to the gastrointestinal tract, and the severe T lymphocyte defects were all factors likely to be related to the sustained impairment of the immune response. The third case shows the visceral involvement of a leishmaniasis case initially confined to the skin region only. The cutaneous leishmaniasis must be differentiated from sporotrichosis, mycobacteriosis, sarcoidosis, syphilis, *lupus vulgaris*, and neoplasms: in general, the histologic examination led to the correct diagnosis. The peri-oral lesions shared characteristics similar to those of the post-kala-azar dermal leishmaniasis (PKDL) [15]. In our case, the diagnosis of PKDL was excluded by the finding of parasites in the bone marrow.

The need of an early clinical advice of the leishmanias diagnosis during the evaluation of HIV febrile patients living in an endemic area is well-known. Nevertheless, unusual presentations of leishmaniasis have to be suspected as differential diagnosis in patients with immunosuppressive conditions, other than HIV infection. We therefore suggest a careful clinical evaluation of immune suppressed patients resident in areas at risk for Leishmania such as the coasts of the Mediterranean Basin. In such patients the occurrence of lymphopenia, anemia, pancytopenia or hypergammaglobulinemia with recurrent febrile episodes or of long-lasting painless ulcerative lesions should alert clinicians to include leishmaniasis in the differential diagnosis.

## Competing interests

The authors of this case presentation declare that they have no financial or non-financial competing interests with regard to the present manuscript

## Authors' contributions

The authors of this case presentation declare that Silvia Pittalis made substantial contributions to acquisition of data and helped to draft the manuscript, Emanuele Nicastri made substantial contributions to draft the manuscript and revised the draft all over the course of submission, Francesco Spinazzola made substantial contributions to acquisition of data and to draft the manuscript, Piero Ghirga made substantial contributions to collection of clinical and biochemical data, Michele De Marco made substantial contributions to acquisition of data, Maria Grazia Paglia carried out the molecular genetic studies, and carried out the immunoassays, Pasquale Narciso conceived of the study, participated in its design and coordination and drafted the manuscript. All authors read and approved the final manuscript.

## Pre-publication history

The pre-publication history for this paper can be accessed here:


